# Adjuvants to the S1-subunit of the SARS-CoV-2 spike protein vaccine improve antibody and T cell responses and surrogate neutralization in mice

**DOI:** 10.1038/s41598-024-80636-3

**Published:** 2024-11-28

**Authors:** William Becker, Khadija Rebbani, Zhijian Duan, Eugene Valkov, Shawn Bryant, Mitchell Ho, Jay A. Berzofsky, Purevdorj B. Olkhanud

**Affiliations:** 1 Vaccine Branch, CCR, NCI, NIH, Bethesda, MD USA; 2 RNA Biology Laboratory, CCR, NCI, NIH, Frederick, MD USA; 3 Antibody Engineering Program, CCR, NCI, NIH, Bethesda, MD USA; 4 Laboratory of Molecular Biology, CCR, NCI, NIH, Bethesda, MD USA; 5 Vaccine Branch, Bldg. 41-Rm D702D (MSC-5062), 41 Medlars Dr., NIH, Bethesda, MD 20892-5062 USA

**Keywords:** Vaccine, SARS-CoV-2, Adjuvants, Variants, Aging, Adjuvants, Immunology, Vaccines, Peptide vaccines

## Abstract

Various public health measures have contained outbreaks of SARS-CoV-2, but concerns remain over the possibility of future surges. Improvements in broadening the vaccine response can stifle new and nascent infections. In this study, we tested the effects of different adjuvant combinations on the immunization of mice with the receptor-binding domain (RBD)-containing the S1-subunit of the spike protein (S1 protein) from SARS-CoV-2 to induce a robust humoral and cellular immune response. We showed that subcutaneous immunization of S1 protein co-delivered with IL-15 and TLR-ligands (MALP-2, poly I: C, and CpG) or with IL-12 and GM-CSF in DOTAP, or Alum induced significantly high titers of durable antibodies, predominantly IgG1, IgG2a, and IgG2b, that could bind to RBD, S1-subunit, and the full-length ectodomain of SARS-CoV-2 spike protein in sera compared to the immunization with S1 protein alone in both B6 wild-type (WT) and the K18-hACE2 transgenic mice. In addition, immunization with S1 protein co-delivered with IL-15 and TLR-ligands induced antibody responses against S1 protein in aged mice, and sera from younger mice reduced plaque formation of live SARS-CoV-2, and had effective binding to S1 protein from ten different variants of SARS-CoV-2, including Omicron (B.1.1.529), and greater neutralization activity as early as day 21 post-immunization measured by inhibition of RBD binding to hACE2 than sera from mice immunized with S1 protein alone or co-delivered with Alum. We also identified antibody-binding epitopes using 18-mer peptides with 9-residue overlaps from the S1 protein. CD8^+^ T-cell responses specific to RBD and S1 protein peptide pools were observed up to day 200 post-immunization by tetramer staining. These data show the efficacy of specific immunologically targeted adjuvants for increasing S1 protein immunogenicity in mice and can contribute to more effective vaccines.

**Supplementary Information:**

The online version contains supplementary material available at 10.1038/s41598-024-80636-3.

## Introduction

Since January 2020, over 7 million deaths have occurred worldwide due to SARS-CoV-2 (https://covid19.who.int). Vaccination remains the primary method for viral containment and mitigation of disease severity^1^. A mature SARS-CoV-2 has four structural proteins: envelope (E), membrane (M), nucleocapsid (N), and spike that can be target antigens to induce neutralizing antibody and T-cell responses^2^. The spike protein is expressed on the surface of the virus, and contains the receptor-binding domain (RBD, comprising a 193 aa region that spans from aa residue from 318 to 510), which mediates viral entry into target cells, through the host angiotensin-converting enzyme 2 (ACE2) receptor^3^. To block this interaction, most vaccines induce immunity against SARS-CoV-2 spike protein^4^.

Despite the unprecedented speed and success of spike-based mRNA vaccines against SARS-CoV-2 at the beginning of the pandemic^5^, immunizations with recombinant protein vaccines (subunit or full-length), viral vaccines (replicating and non-replicating), and nucleic acid vectored vaccines (DNA and mRNA), and passively transferring monoclonal neutralizing antibodies against the spike protein are available to protect against infection and worsening of disease^1,6^. However, over the last four years, as waning efficacy of initial vaccines occurs, combined with long lived virus in some individuals, SARS-CoV-2 has mutated, diversifying its viral lineage and spawning variants of concern (VOC)^7,8^. The emergence and prevalence of VOCs underscore the need for improved vaccines that can provide broadened immunity to new, nascent, and future VOCs^9,10^. One way to augment protein-based vaccines is with potent adjuvants that improve the magnitude and durability of humoral and cellular immunogenicity^11^.

Based on our previous studies using a combination of different adjuvants in HIV and cancer vaccines^12,13^, we investigated the effects of different adjuvants on the immunization of mice with the receptor-binding domain (RBD)-containing S1-subunit of the spike protein (S1 protein) from SARS-CoV-2 to enhance vaccine responses in mice. In this study, we evaluated immunization of S1 protein co-delivered in DOTAP with IL-15 and TLR-ligands (TLR-Ls) (MALP-2, poly I: C, and CpG) or with IL-12 and GM-CSF, or Alum (aluminum salts), using S1 protein alone as a control. We found that mice immunized with S1 protein co-delivered with IL-15 and TLR-Ls provided the best immunity against SARS-CoV-2 compared to the other adjuvant combinations tested, eliciting significantly higher titers of durable antibody that could bind to RBD, S1, and the full-length ectodomain of SARS-CoV-2 spike protein in sera compared in both B6 wild-type (WT) and the K18-hACE2 transgenic mice that express human ACE2 (hACE2). Aged mice showed a response to immunization with S1 protein co-delivered with IL-15 and TLR-Ls whereas no response was seen in aged mice immunized with S1 protein alone. Moreover, sera from mice immunized with S1 protein co-delivered with IL-15 and TLR-Ls had effective binding to S1 protein from ten different variants of SARS-CoV-2, including Omicron and its subvariants (B.1.1.529 and BA.4/BA.5). Sera from this adjuvant combination showed greater neutralization activity as early as day 21 post-immunization measured by inhibition of RBD binding to hACE2 than sera from mice immunized with S1 protein alone or co-delivered with Alum and greater live virus neutralization at day 60 post-immunization. CD8^+^ T-cell responses specific to RBD and S1 protein peptide pools were seen up to day 200 post-immunization by tetramer staining. These data show the efficacy of specific immunologically targeted adjuvants for increasing S1 protein immunogenicity in mice and can contribute to the design of more effective vaccines.

## Materials and methods

### Mice

8-12-week-old female C57BL/6 (RRID: IMSR_JAX:000664**)** mice were purchased from Charles River and K18-hACE2 [B6.Cg-Tg(K18-ACE2)2Prlmn/J; RRID: IMSR_JAX:034860] transgenic mice were purchased from Jackson Laboratories. All animals were subsequently bred and housed at the NIH Animal facility. All animal procedures reported in this study that were performed by NCI-CCR affiliated staff were approved by the NCI Animal Care and Use Committee (ACUC) and in accordance with federal regulatory requirements and standards. Mice were euthanized via CO2 inhalation. All components of the intramural NIH ACU program are accredited by AAALAC International. All animal reporting was conducted in accordance with ARRIVE guidelines.

### Reagents

All recombinant S1 proteins from different variants of SARS-CoV-2 were purchased from Sino Biological. Recombinant SARS-CoV-2 RBD protein (Accession no.: QHD43416.1) was purchased from Leinco Technologies. The ectodomain of SARS-CoV-2 spike protein was designed and purified in the lab. It comprises residues M1-P1213 based on the viral isolate sequence to GenBank ID MN908947.3. A signal peptide (MFVFLVLLPLVS) is located at the N-terminus and a double StrepII (WSHPQFEK) tag is located at the C-terminus.

### Ectodomain synthesis

The gene encoding the ectodomain from the Spike protein of SARS-CoV-2 was synthesized commercially by GeneWiz (Azenta Life Sciences) with optimized codon usage for Spodoptera frugiperda and inserted into a pLIB plasmid vector (Addgene #80610) between BamHI and HindIII sites. The construct comprised residues M1–P1213 based on the viral isolate sequence corresponding to GenBank ID MN908947.3. A signal peptide (MFVFLVLLPLVS) was fused at the N-terminus, and a double StrepII (WSHPQFEK) tag is at the C-terminus. A single alanine residue substituted the S1/S2 polybasic furin cleavage site (residues R682–R685). In addition, the K986–V987 pair in the C-terminal S2 fusion domain was replaced by rigidifying prolines to stabilize the protein in the pre-fusion^14^. The construct does not include the foldon motif from bacteriophage T4 fibritin used in some studies, as we have observed that the native Spike trimerizes stably in solution. The V0 recombinant baculovirus was generated using the MultiBac system^15^ in adherent cells. The initial low-titer V0 virus was amplified to high-titer V1 generation in suspension state. The protein was produced via a secretion pathway in Sf21 insect cells cultured in SF900II serum-free media (Thermo Fisher Scientific). The Sf21 cells were grown in suspension to a density of 2 × 10^6^ cells/ml at 27 °C and infected with the V1 baculovirus at a ratio of 1 ml of virus per 100 ml of culture. Four days after infection, cells were harvested by centrifugation at 1000 g for 10 min at 4 °C. The pellet was discarded, and the supernatant was clarified by a second centrifugation step at 5000 g for 10 min at 4 °C. BioLock biotin blocking solution (IBA Life Sciences) was added to the clarified solution at a ratio of 2.5 ml per liter. The supernatant was then applied at a flow rate of 1 ml/min to a 1 ml StrepTrap XT column (Cytiva) equilibrated in phosphate-buffered saline solution (PBS). After washing for several column volumes, the bound fraction was eluted in PBS supplemented with 2.5 mM desthiobiotin (Sigma Aldrich). Following affinity capture, the protein was further polished using size-exclusion chromatography on an S200 26/60 column (Cytiva) equilibrated in PBS and eluted at 2.5 ml/min. The protein eluted as a symmetric peak with a molecular weight corresponding to a trimer (~ 400 kDa). Peak fractions were analyzed for purity by SDS-PAGE and concentrated to ~ 0.6 mg/ml using Amicon centrifugal concentrators with a 100 kDa molecular weight cut-off. The concentrated protein was aliquoted in 25 µl aliquots and flash-frozen in liquid nitrogen with storage at -80 °C or stored on dry ice until use.

### Mouse immunization

Female B6 WT or hACE2 transgenic mice aged 8–12 weeks (young) or over 18 months (aged) were immunized subcutaneously with 10 µg of S1 protein (Accession no.: QHD43416.1, YP_009724390.1, Val16-Arg685, Sino Biological) co-delivered in 20 µg of DOTAP (Roche) with 15 µg of IL-15 (Peprotech) and TLR-Ls (0.1 µg of MALP-2 (Enzo Life Sciences), 50 µg of poly I: C (InVivoGen), and 5 µg of CpG-ODN 1826 (InVivoGen)) or with 5 µg of IL-12p70 (Biolegend) and 5 µg of GM-CSF (Biolegend), or with 50 µg of Alum (Thermo Scientific) and alone as a control group. Concentrations of adjuvants are based on previous work including:^12,17,19^. (Mice were immunized subcutaneously in the right flank three times with two-week intervals. Sera were collected and analyzed two weeks after each immunization and at days 75 and 200 post-immunization. In addition, spleen cells were collected and analyzed at days 42, 75, and 200 post-immunization.

### ELISA

96-well Nunc MaxiSorp ELISA plates were coated with 50 µl/well of recombinant protein indicated at a concentration of 5 µg/ml in PBS overnight at 4 °C. The coating buffer was removed, and plates were washed and incubated for 1 h at RT with 200 µl blocking solution. Serum was diluted 1:5,000 to 1:100,000 in assay diluent, and 100 µl of diluted serum was added for 1 h at RT. Plates were washed three times with wash buffer, and 50 µl of mouse IgG, IgG1, IgG2a, or IgG2c-specific HRP-conjugated secondary antibody (Novus Biologicals) was added to each well. After 1 h of incubation at RT, plates were washed three times with wash buffer and were developed with 100 µl of TMB Substrate Reagent Set, and the reaction was stopped after 15 min by the addition of 2 N sulfuric acid. Plates were then read at a wavelength of 450 nm and 570 nm. Anti-SARS-CoV-2 (2019-nCoV) S1 neutralizing antibody (Sino Biological) was used as reference standards to calculate to create the standard curve and quantify antibody level.

### Isolation of mononuclear cells and flow cytometry

Spleen cells were minced using a gentleMACS tissue dissociator (Miltenyi Biotec) followed by 70 μm filtrations to obtain a single-cell suspension. MNCs from each mouse were cultured in culture media (RPMI1640 supplemented with 10% heat-inactivated FBS, penicillin-streptomycin, L-glutamine, HEPES, NEAA, sodium pyruvate, and 2ME) in 96-well plates at 37 °C for 6 h in the presence of overlapping peptide pool mixture of RBD (15 mer peptides with 11-residue overlaps, PepMix SARS-CoV-2 S-RBD, JPT) or S1 (15 mer peptides with 11-residue overlaps, PepTivator SARS-CoV-2 S1, Miltenyi Biotec) proteins of SARS-CoV-2 and brefeldin A (BD Biosciences). The final concentration of both peptide libraries was used at 0.1 µg/ml. Following stimulation, cells were washed with PBS and incubated on ice with Fc block and Live/Dead fixable blue dead cell stain (Invitrogen) for 20 min at RT. Cells were then labeled for surface markers on ice for 30 min, fixed/permeabilized using BD Cytofix/Cytoperm solution kit (BD Biosciences) according to the manufacturer’s instructions. Cells were then washed and labeled with an intracellular antibody cocktail. All flow cytometry data were acquired on a BD FACS Symphony A5 and were analyzed using FlowJo Software.

### Antibodies

The following monoclonal antibodies were used: rat anti-mouse CD45 (clone 30-F11), CD3e (clone 145-2C11), CD4 (clone RM4-5), CD8 (clone 53 − 6.7), IFNγ (clone XMG1.2), TNFα (clone MP6-XT22), all from BD Biosciences and H-2 K(b) SARS-CoV-2 S (539–546, VNFNFNGL) tetramer (NIH Tetramer Core Facility).

### Overlapping peptides used in antibody response

The peptide library (comprising 74 peptides in total) covering the spike S1 subunit was synthesized by GenScript, with a purity exceeding 95%. Beginning from the residue following the signal sequence, 18-mer peptides were synthesized, each with a 9-residue overlap with each other sequentially. All peptides were dissolved and prepared according to the instruction provided by GenScript.

### Surrogate neutralization assay

ImmunoRank Neutralization Micro-ELISA (Leinco Technologies) assay was used as a surrogate neutralization following the manufacturer’s instruction. The assay measures all immunoglobulin subclasses in serum capable of binding to the RBD and neutralizing or blocking the interaction between the RBD and hACE2 receptor. In brief, 1:10 diluted serum samples collected from immunized mice were incubated with soluble RBD protein conjugated to HRP for 30 min in RT, and the mixture was then added to wells coated with recombinant hACE2 protein for 30 min at 37 °C. After the incubation period, the wells were washed to remove unbound samples, and TMB is added to quantify the concentration of antibodies specific to RBD. The sample neutralization index (SNI%) expressed as percent neutralization for each sample was calculated using the following formula, SNI% = [ 1 – (Sample OD450 / Negative Control OD450) ] / [ 1 – (Positive Control OD450 / Negative Control OD450) ] x 100. Samples with SNI% < 20 are considered negative or have a non-detectable level of RBD-specific antibody.

### PRNT

Plaque Reduction Neutralization Test (PRNT) was performed in duplicate using Vero E6 cells (ATCC, CRL-1586) and 30 PFU challenge titers of SARS-CoV-2 (USA-WA1/2020 and Omicron BA.1 strain). Serum samples were tested at a starting dilution of 1:20 and were serially diluted 3-fold up to final dilution of 1:14,580. After serum incubation with 30 PFU of SARS-CoV-2 for 1 h at 37 °C, serial dilutions of virus-serum mixtures were added onto Vero E6 cell monolayers. Cell culture medium with 1% agarose was added to the cells, following incubation for 1 h at 37 °C with 5% CO2. The plates were fixed and stained after 3 days of culture. Antibody titer ID50 and ID90 were defined as the highest serum dilution resulting in 50% and 90% reduction of plaques, respectively.

### Statistical analysis and illustration

Comparison of data between groups was performed using two-sided Mann-Whitney tests or a Two-way ANOVA with Tukey’s multiple comparisons test where appropriate. Correlations were assessed by two-sided Spearman rank-correlation tests. Results are expressed as mean and SEM. All statistical tests were calculated using GraphPad Prism (GraphPad software). *p* values of < 0.05 were considered statistically significant (*, *p* < 0.05; **, *p* < 0.005; ***, *p* < 0.0005; ****, *p* < 0.00001).

## Results

### Cytokine and chemokine adjuvant combination enhances the humoral and T cell immune response of S1 vaccination

**Fig. 1:**
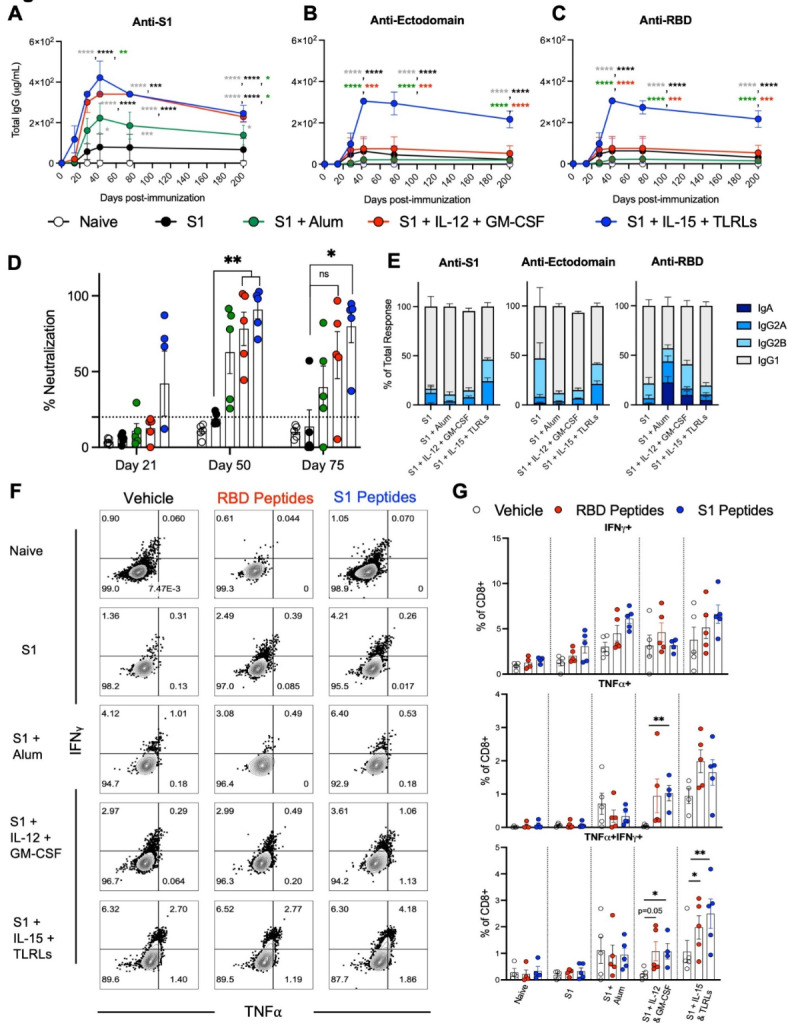
Cytokine and TLR-ligand adjuvant combination enhances the humoral and T cell immune response of S1 protein immunization. Immunization schedule: B6 (WT) mice were immunized with priming on day 0 and two booster doses, at two-week intervals. Mice were immunized with S1 protein alone (black) or co-delivered with Alum (green), IL-12 and GM-CSF (red), or IL-15 and TLR-Ls (blue) in DOTAP. Serum samples were collected at days 14, 28, 42, 75, and 200 post-immunization. Spleen cells were collected at day 200 post-immunization. Humoral immune responses were assessed following immunization by analysis of total serum IgG responses to (**A**) S1, (**B**) ectodomain, or (**C**) RBD of SARS-CoV-2 spike protein over time evaluated by ELISA. (**D**) Serum antibody neutralization potencies at days 21, 50, and 75 are presented as SNI% in 1:30 dilutions of sera against RBD-hACE2 binding. The dashed horizontal line corresponds to the lower limit of detection (LLOD = 20%) of the assay used. The data are represented as the mean ± SEM and represent at least two independent experiments. *p* values are shown for entirety of study using one (**D**) or two-way ANOVA (**A**-**C**) test with post hoc Tukey’s multiple comparisons; *, *p* < 0.05; **, *p* < 0.01; ***, *p* < 0.001; ****, *p* < 0.0001. (**E**) Day 45 post-immunization serum samples were subjected to ELISAs for S1, RBD, or ectodomain of spike protein reactivity and antibody isotypes were assessed and normalized to total antibody response. (**F** and **G**) Cellular immune responses were assessed at day 200 post-immunization by stimulating spleen cells ex vivo with either vehicle control or overlapping peptide pools comprising the RBD or S1 proteins, all peptides at 0.1 µg/ml, for 6 h and then assessed by flow cytometry for intracellular production of IFNγ and TNFα. (**F**) Representative flow cytometry contour plots and (**G**) graphed values of individual mice. *, *p* < 0.05; **, *p* < 0.01; ***, *p* < 0.001; ****, *p* < 0.0001 determined using a paired two-tailed Student’s *t*-test.

We first assessed the humoral responses to SARS-CoV2 S1 protein vaccination with different combination of adjuvants. Binding antibody responses against S1 protein, RBD protein located in the S1 subunit, and the full-length ectodomain of SARS-CoV-2 spike protein were assessed by ELISA in 8-week-old female B6 WT mice by three subcutaneous immunizations consisting of 10 µg of S1 protein with or without adjuvants, co-delivery of IL-15 and TLR-Ls or IL-12 and GM-CSF in DOTAP, or Alum, separated by two-week intervals. Single-dose (prime) S1 protein immunization co-delivered with IL-15 and TLR-Ls induced the highest titers of antibodies specific to S1, ectodomain, and RBD of spike protein, which remained high at days 75 and 200 post-immunization (Fig. 1A and C where y-axis is in log10 scale). It is important to note among the groups, only the S1 protein co-delivered with IL-12 and GM-CSF group had similar but lower response to the S1 protein co-delivered with IL-15 and TLR-Ls group against S1 protein, the immunogen, but not against ectodomain or RBD proteins. Additionally, the S1 protein co-delivered with Alum group exhibited a greater response than the S1 protein alone.

Surrogate virus neutralization activity was assayed by measuring the ability of antibodies induced by S1 protein immunization to block binding of RBD to hACE2. Sera from mice immunized with S1 protein co-delivered with IL-15 and TLR-Ls showed potent surrogate neutralization activity as early as day 21 post-immunization that remained significantly higher than S1 protein alone through days 50 and 75 post-immunization. S1 protein co-delivered with Alum or IL-12 and GM-CSF, however, elicited surrogate neutralization superior to S1 protein alone at day 50 post-immunization, but neutralization activity waned by day 75 post-immunization. Mice immunized with S1 protein alone showed no neutralization activity at any time points we tested, suggesting that S1 protein alone is not immunogenic and co-delivery with adjuvants is required to enhance immunogenicity in S1 protein immunization in mice (Fig. 1D).

Next, we evaluated the levels of S1-specific immunoglobulins, IgG1, IgG2a, IgG2b, and IgA, among which IgG1 and IgG2a are surrogates of Th2 and Th1 responses, respectively. Among IgG subclasses, all adjuvants elicited high levels of S1-specific IgG1 and lower levels of IgG2a and IgG2b, suggesting a Th2-biased response (Fig. 1E). The IgG-subset profile was similar across the reactivities we tested, and no S1-specific IgA was detectable in the serum at any timepoint assayed (Fig. 1E). In addition, we measured cytokine levels pertaining to T helper phenotype and activation status in sera collected from immunized mice at days 14 and 42 post-immunization. The only measurable differences observed were increases in TNFα and IL-2 in the group of mice immunized with S1 protein co-delivered with IL-12 and GM-CSF adjuvant combination at day 42 post-immunization (Supplementary Fig. 1A and 1B).

We next determined memory T cell responses against the spike protein via intracellular cytokine staining. Day 200 post-immunization, spleen cells were harvested and stimulated ex vivo with a peptide pool (a single pool of overlapping peptides) covering either the RBD only or S1 protein for 6 h. Splenocytes from mice immunized with S1 protein co-delivered with IL-15 and TLR-Ls had more CD8^+^ T cells secreting IFNγ, TNFα, or both cytokines, after re-stimulation, than any other group. The splenic CD8^+^ T cells from the group immunized with S1 protein co-delivered with IL-12 and GM-CSF also had an increase in TNFα and double positive IFNγ^+^TNFα^+^ cells after restimulation with S1 or RBD protein peptide pools (Fig. 1F and G).

### Enhancement of humoral and cellular response to S1 protein immunization by adjuvants is specific

**Fig. 2:**
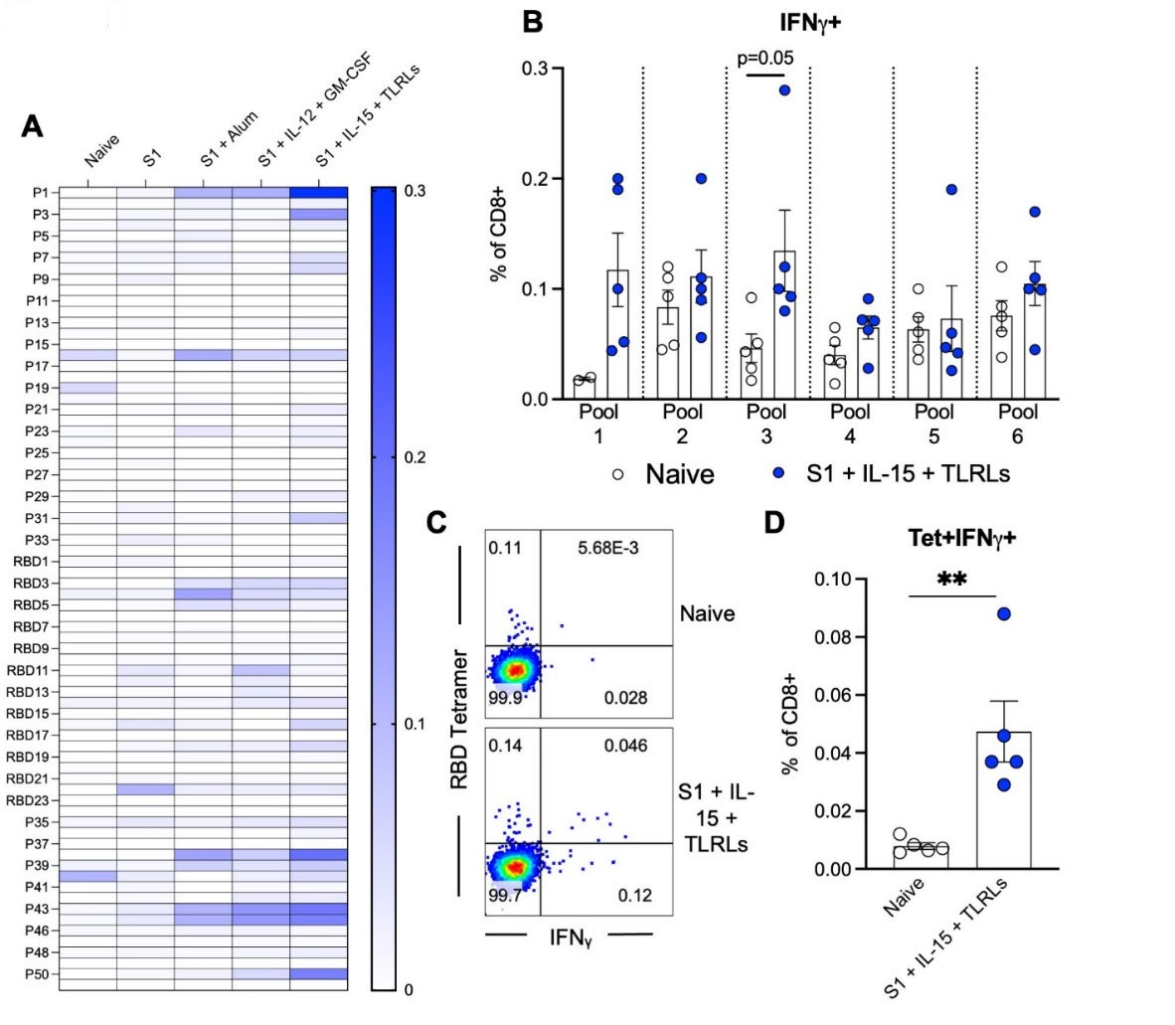
Enhancement of humoral and cellular response to S1 protein immunization by adjuvants is specific. B6 (WT) mice were immunized with priming on day 0 and two booster doses, each at two-week intervals. Mice were immunized with S1 protein alone, or co-delivered with Alum, IL-12 and GM-CSF, or IL-15 and TLR-Ls in DOTAP. Serum samples were collected at day 45 post-immunization and subjected to ELISAs for individual peptides from S1 and RBD proteins, with responses shown as a heat map (**A**). Spleen cells collected at day 200 post-immunization were stimulated ex vivo with pooled groups of peptides from S1 and RBD proteins for 6 h before staining for intracellular production of IFNγ by total CD8^+^ T cells (**B**) was assessed. (**C**) Representative flow cytometry dot plot of RBD-tetramer^+^ and IFNγ^+^ CD8^+^ T cells deriving from spleens of naïve mice or mice immunized with S1 protein co-delivered with IL-15 and TLR-Ls and ex vivo exposed to pool 3 for 6 h. (**D**) Graphed individual values. The data are represented as the mean ± SEM and represent 2 independent experiments. *, *p* < 0.05; **, *p* < 0.01; ***, *p* < 0.001; ****, *p* < 0.0001 determined using an unpaired two-tailed Student’s *t*-test.

To better assess the specificity of the antibody response induced by our adjuvant combinations, 18-mer peptides with 9-residue overlaps comprising the S1 protein (Supplementary Fig. 2A) were tested against serum from naive or immunized mice. The highest response to these peptides was seen in mice immunized with S1 protein co-delivered with IL-15 and TLR-Ls, specifically at peptides 1, 38, 44, 45, and 50, with mice immunized with S1 protein co-delivered with IL-12 and GM-CSF or Alum having less of a response but still an increase compared to the mice immunized with S1 protein alone (Fig. 2A). Due to the higher antibody levels induced by S1 protein co-delivered with IL-15 and TLR-Ls compared to S1 protein alone, higher consistent surrogate neutralization activity, and a greater T cell response to ex vivo restimulation with RBD and S1 protein peptide pools (Fig. 1); we focused on co-delivery of IL-15 and TLR-Ls as an adjuvant combination in subsequent assays to dissect its potency more thoroughly. We narrowed the focus of the T cell response by ex vivo stimulation of splenocytes from naïve mice or mice immunized with S1 protein co-delivered with IL-15 and TLR-Ls for 6 h with six non-overlapping peptide pools that comprise the entire S1 protein (Supplementary Fig. 1A). Splenocytes from mice immunized with S1 protein co-delivered with IL-15 and TLR-Ls stimulated with pool 3 had significantly more CD8^+^ T cells secreting IFNγ than the naïve mice (Fig. 2B). Pool 3 has non-overlapping peptides that are a majority of the RBD protein. Concordantly, stimulating splenocytes from mice immunized with S1 protein co-delivered with IL-15 and TLR-Ls resulted in greater numbers of CD8^+^ T cells double positive for IFNγ and MHC I tetramer containing the peptide binding groove of RBD (Fig. 2C and D).

### Immune response to adjuvanted S1 protein vaccine in K18-hACE2 mice and aged mice

**Fig. 3:**
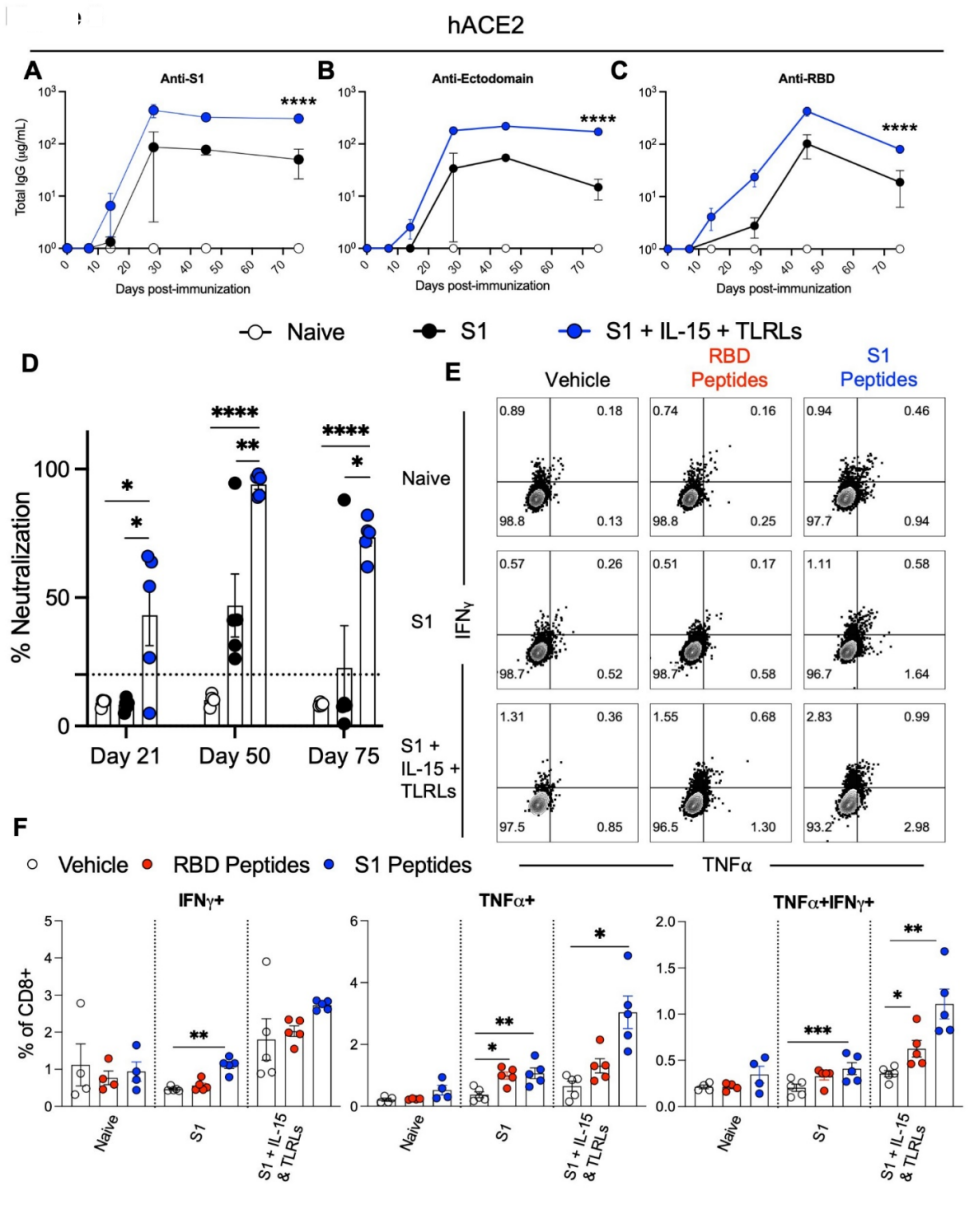
Immune response to adjuvanted S1 protein vaccine in hACE2 mice. K18-hACE2 [B6.Cg-Tg(K18-ACE2)2Prlmn/J] transgenic mice were immunized with S1 protein alone or S1 protein co-delivered with IL-15 and TLRLs. Serum samples were collected at days 14, 28, 45, and 75 post-immunization, and spleen cells were collected at day 200 post-immunization. Humoral immune responses were assessed following immunization by analysis of total serum IgG responses to (**A**) S1, (**B**) ectodomain, or (**C**) RBD of SARS-CoV-2 spike protein over time evaluated by ELISA. (**D**) Serum antibody neutralization potencies at days 21, 50, and 75 are presented as SNI% in 1:30 dilutions of sera against RBD-hACE2 binding. The dashed horizontal line corresponds to the lower limit of detection (LLOD = 20%) of the assay used. The data are represented as the mean ± SEM and represent at least two independent experiments. *p* values are shown for entirety of study using one (**D**) or two-way ANOVA (**A**-**C**) test with post hoc Tukey’s multiple comparisons; *, *p* < 0.05; **, *p* < 0.01; ***, *p* < 0.001; ****, *p* < 0.0001. Cellular immune responses were assessed at day 75 post-immunization by stimulating spleen cells ex vivo with either vehicle control or overlapping peptide pools comprising the RBD or S1 proteins, all peptides at 0.1 µg/ml, for 6 h and then assessed by flow cytometry for intracellular production of IFNγ and TNFα in CD8^+^ T cells. (**E**) Representative flow cytometry contour plots and (**F**) graphed values of individual mice. *, *p* < 0.05; **, *p* < 0.01; ***, *p* < 0.001; ****, *p* < 0.0001 determined using a paired two-tailed Student’s *t*-test.

To address whether immunization of S1 protein co-delivered with IL-15 and TLR-Ls display differences in the immune response in B6 WT mice versus K18-hACE2 transgenic mice which have the human ACE2 receptor for RBD (which is lacking in mice) of spike protein, we tested our adjuvant combination against S1 protein alone as was done previously. Humoral responses to S1 protein co-delivered with IL-15 and TLR-Ls was consistent in K18-hACE2 with what was seen in WT mice, such that serum levels of IgG against S1, ectodomain, and RBD of spike protein were significantly higher with S1 protein co-delivered with IL-15 and TLR-Ls than with S1 protein alone as early as day 14 post-immunization and remained several log higher than in animals immunized with S1 protein alone until day 75 post-immunization (Fig. 3A and C). Indeed, the antibody responses were detectable nearly a week earlier after immunization with S1 protein co-delivered with IL-15 and TLR-Ls (Fig. 3A and C). Similarly, surrogate neutralization activity of sera from mice immunized with S1 protein co-delivered with IL-15 and TLR-Ls was significantly higher at days 21, 50, and 75 post-immunization compared to mice immunized with S1 protein alone (Fig. 3D). The T cell response at day 75 post-immunization was also higher in mice immunized with S1 protein co-delivered with IL-15 and TLR-Ls (Fig. 3E and F).

**Fig. 4:**
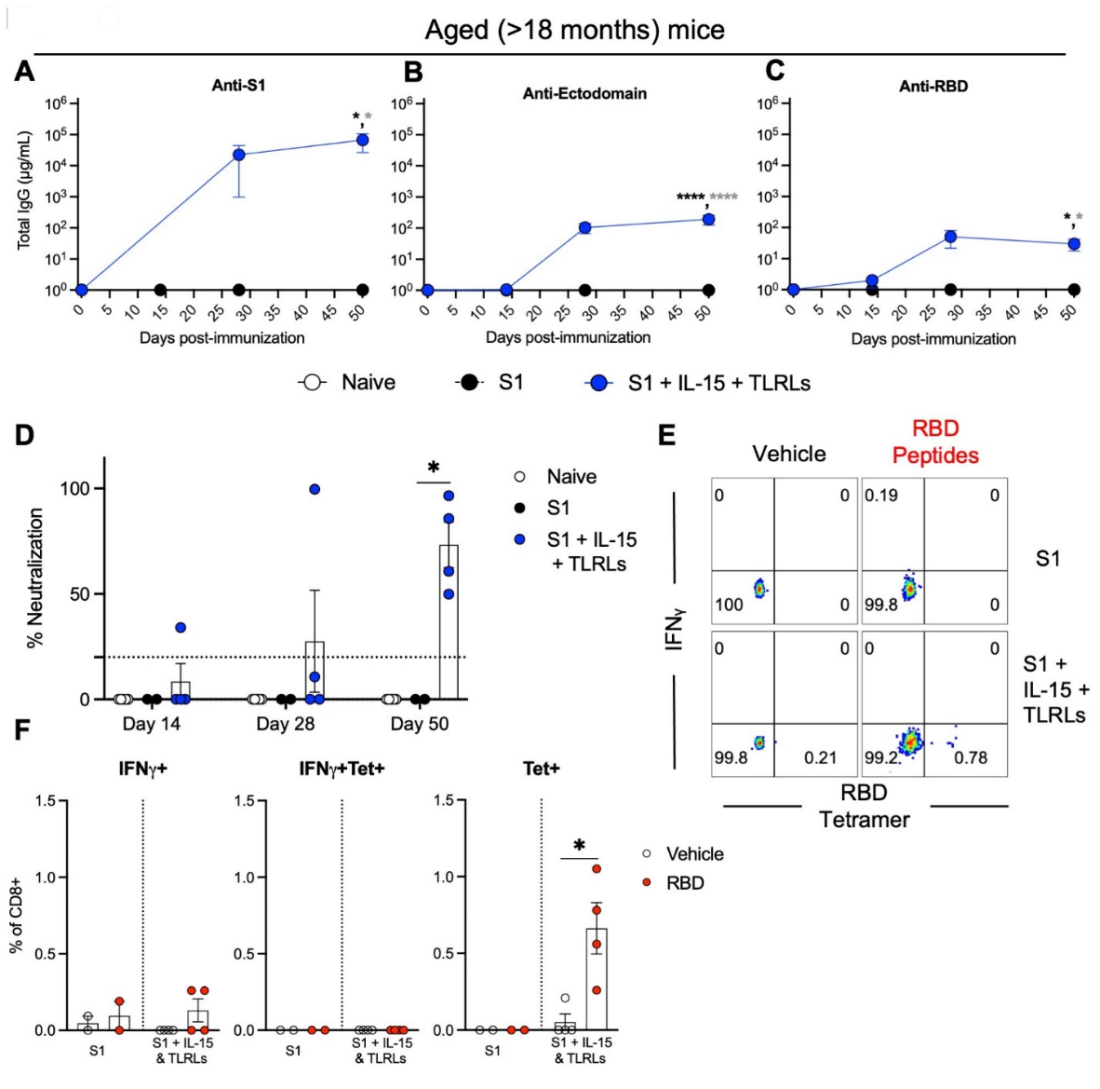
Adjuvanted S1 protein vaccine efficacy in aged mice. Aged (> 18 months old) B6 (WT) mice were immunized with S1 protein alone or S1 protein co-delivered IL-15 with TLR-Ls. Humoral immune responses were assessed following immunization by analysis of total serum IgG responses to (**A**) S1, (**B**) ectodomain, or (**C**) RBD of SARS-CoV-2 spike protein over time evaluated by ELISA. (**D**) Serum antibody neutralization potencies at days 14, 28, and 50 are presented as SNI% in 1:30 dilutions of sera against RBD-hACE2 binding. The dashed horizontal line corresponds to the lower limit of detection (LLOD = 20%) of the assay used. The data are represented as the mean ± SEM and represent at least two independent experiments. *p* values are shown for entirety of study using one (**D**) or two-way ANOVA (**A**-**C**) test with post hoc Tukey’s multiple comparisons; *, *p* < 0.05; **, *p* < 0.01; ***, *p* < 0.001; ****, *p* < 0.0001. Cellular immune responses were assessed at day 75 post-immunization by stimulating spleen cells ex vivo with either vehicle control or overlapping peptide pools comprising the RBD protein at 0.1 µg/ml of all peptides for 6 h and then assessed by flow cytometry for RBD-tetramer^+^ CD8^+^ T cells and intracellular production of IFNγ in CD8^+^ T cells. (**E**) Representative flow cytometry contour plots and (**F**) graphed values of individual mice. *, *p* < 0.05; **, *p* < 0.01; ***, *p* < 0.001; ****, *p* < 0.0001 determined using a paired two-tailed Student’s *t*-test.

We next compared the immune response to immunization in aged (>18 months old) B6 WT mice. Unlike in younger mice (8–12 weeks old), aged mice immunized with S1 protein alone elicited no detectable antibodies against S1, ectodomain, or RBD of spike protein; however, adding the adjuvant combination of IL-15 and TLR-Ls to S1 protein immunization induced high titers of antibodies against S1, ectodomain, and RBD of spike protein in aged mice (Fig. 4A and C); however, these antibodies were not detectable until after the first boost on day 14 post-immunization, nor were the levels of antibodies at sufficient levels to provide surrogate neutralization activity until after the second boost on day 28 post-immunization (Fig. 4D). Splenocytes from aged mice immunized with S1 protein co-delivered with IL-15 and TLR-Ls at day 50 post-immunization were stimulated with a pool of overlapping RBD peptides for 6 h ex vivo, revealing a significant increase in the amount of RBD tetramer^+^ CD8^+^ T cells induced after peptide re-stimulation compared to media control, and this effect was not seen in aged mice immunized with S1 protein alone (Fig. 4E and F).

### Co-delivery of IL-15 and TLR-Ls provides the best humoral protection against live SARS-CoV-2 virus and variants of concern

**Fig. 5:**
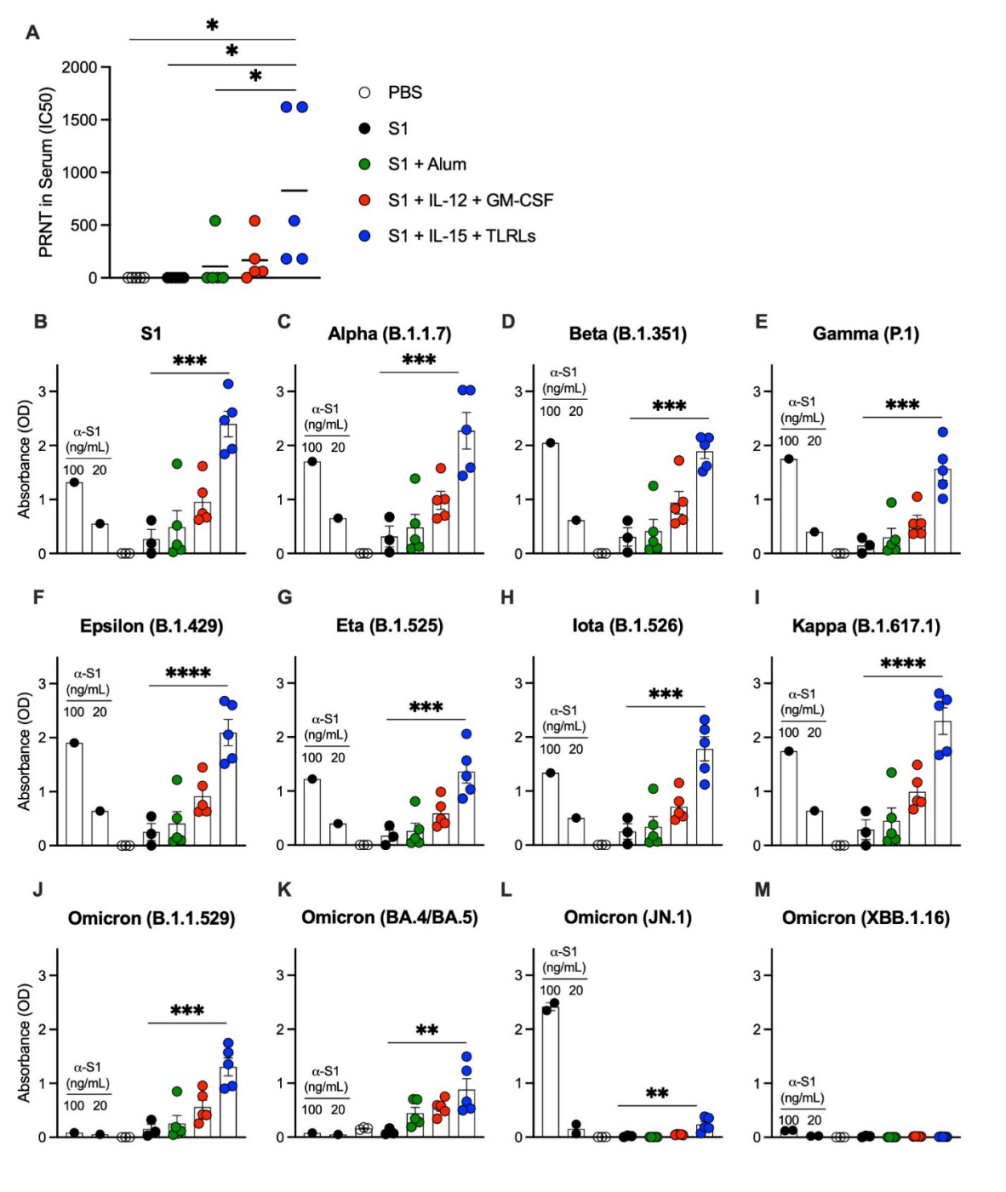
Immunization of S1 protein co-delivery of IL-15 with TLR-Ls provides the best humoral protection against live SARS-CoV-2 virus and variants of concern. B6 (WT) mice were immunized with S1 protein alone (black) or co-delivered with Alum (green), IL-12 and GM-CSF (red), or IL-15 and TLR-Ls (blue) in DOTAP. Serum samples from day 60 post-immunization were incubated with 30 PFU of SARS-CoV-2 for 1 h at 37 °C; serial dilutions of virus-serum mixtures were added onto Vero E6 cell monolayers and plates were fixed and stained after 3 days of culture. (**A**) PRNT (Plaque Reduction Neutralization Test) antibody titer ID50 was defined as the highest serum dilution resulting in 50% reduction of plaques. Serum samples from day 60 post-immunization and anti-S1 positive control antibodies were subjected to ELISAs (read as absorbance) for reactivity against SARS-CoV-2 variants, including WT (Wuhan) and variants of concern (**B**-**M**). The data are represented as the mean ± SEM and represent one (**A**) or two (**B**-**M**) independent experiments. *p* values are shown for entirety of study using one-way ANOVA test with post hoc Tukey’s multiple comparisons; *, *p* < 0.05; **, *p* < 0.01; ***, *p* < 0.001; ****, *p* < 0.0001.

We next tested whether antibodies induced by our vaccine adjuvant combinations can neutralize live SARS-CoV-2 *in vitro.* Only serum from the group immunized with S1 protein co-delivered with IL-15 and TLR-Ls could reduce plaque formation and increase the IC50 to levels significantly higher than naïve mice or mice immunized with S1 protein alone or co-delivered with Alum (Fig. 5A). Considering the increasing prevalence of variants of SARS-CoV-2, we tested how serum from our groups of interest would react against S1 proteins from eleven different variants of concern and WT (Wuhan) variant of SARS-CoV-2. For the variants tested, only serum from mice immunized with S1 protein co-delivered with IL-15 and TLR-Ls provided an antibody response significantly better than S1 protein alone to every variant tested, even to Omicron variants B1.1.529 and BA.4/BA.5, which were not reactive to the commercial anti-S1 antibody we tested (Fig. 5B and K). Omicron variant JN.1 was responsive to commercial anti-S1 antibody, and weakly reactive to sera of mice immunized with S1 protein co-delivered with IL-15 and TLR-Ls compared to the other groups, but neither the anti-S1 antibody nor immunized mouse sera were reactive against Omicron variant XBB.1.16 (Fig. 5L and M). There was no detectable antibody response from the groups tested against S2-subunit of the SARS-CoV-2 spike protein, which was not part of the vaccine (Supplementary Fig. 2B).

## Discussion

The decline in immunity generated by the initial vaccines to SARS-CoV-2, especially as it pertains to the spread of Omicron, represents the next challenge in managing the spread of SARS-CoV-2. The Pfizer-BioNTech mRNA vaccine, one of the most efficacious vaccines against SARS-CoV-2 and one of the first to be used internationally, declined in efficacy from greater than 90–45% against Omicron in the ten weeks following the second booster (third immunization), demonstrating the rapidly waning immunity against the leading VOC^10^. Here, we show how amongst the adjuvants tested for augmenting a S1 protein of WT (Wuhan) SARS-CoV-2 spike protein vaccine: co-delivered with Alum, IL-12 and GM-CSF or IL-15 with TLT-Ls in DOTAP, adjuvanting a S1 protein vaccine co-delivered with IL-15 and TLR-Ls can provide the broadest and most robust cellular and humoral responses against SARS-CoV-2, including Omicron and its subvariants, B1.1.529, JN.1 and BA.4/BA.5. We found this adjuvant combination provided a long-lasting T cell response up to 200 days post primary immunization, and that the serum antibody response induced by this combination neutralized RBD binding to hACE-2, and live virus neutralization through PRNT assay 60 days post primary immunization.

Although Alum and the combination of IL-12 and GM-CSF in DOTAP are strong adjuvants for inducing a potent T cell response to viral challenge^12^, the combination of IL-15 and TLR-Ls in DOTAP activating TLRs 2, 3 and 9 provided the best anti-SARS-CoV-2 response. The combination of IL-15 and TLR-Ls in DOTAP synergize via TLR-L stimulation of the innate immune system through PRRs, which recognize PAMPs^16^ and complementary activation of MyD88 and TRIF dendritic cell pathways^17^, and adding IL-15 stimulates the adaptive immune response and provides long-lasting CD8^+^ T cells^18,19^, with antigen specificity directed at the co-delivered peptide. IL-15 is being used in trials for cancer and autoimmunity to increase NK cell and CD8^+^ T cell numbers^20^; however no trials are underway at the time of the writing to indicate using IL-15 with vaccines against SARS-CoV-2, despite transient increases of circulating IL-15 levels early after boost being a potential prognostic indicator of mRNA vaccine-mediated effective humoral immune response to SARS-CoV-2^21^. TLR-Ls are being tested in clinical trials as both drug targets^22^, adjuvants for SARS-CoV-2, and other viral vaccines^23,24^.

Another finding of our study was that co-delivery of IL-15 and TLR-Ls in DOTAP can be an effective adjuvant combination for enhancing immunity against SARS-CoV-2 in aged (> 18 months old) mice. Notably, vaccine responses vary considerably according to the recipient’s age and sex, with more potent effects in females than males and in younger people than the elderly^25,26^. Aged mice immunized against the original SARS-CoV seemed to display increased immunopathology rather than protection^27^. Thus, our vaccine platform’s ability to stimulate a strong immune response in aged mice has clinical implications for vaccinating the elderly population that is most susceptible to the deleterious effects of SARS-CoV-2 infection, and for whom vaccination with the initial round of mRNA vaccines has shown limited ability to decrease infection with Omicron variants^28^. Besides aging, the other main concern at the current stage of SARS-CoV-2 progression is immune-escape of the dominant strain from immunity induced by the strain first vaccinated against.

Variants of SARS-CoV-2 with multiple immune-escape and infectivity-enhancing mutations, particularly in the spike protein, which facilitates infection of human cells, likely arise after chronic infection^7,29^. These VOCs, especially Omicron and its subvariants, present a growing threat, and concerningly, exhibit antibody evasion properties even in individuals boosted with a WA1/BA.5 bivalent mRNA vaccine^31^. Repeated Omicron infection can override immunity against ancestral SARS-CoV-2^30^, but infection cannot be considered as an intentional or reliable defense, so preventive vaccination remains the best strategy for neutralizing SARS-CoV-2 and all of its variants. There are studies underway trying to make ‘variant proof’ vaccines directed at virus families^32^; however, until those are ready to be implemented, vaccine adjuvants such as co-delivery of IL-15 and TLR-Ls in DOTAP represent a simple and effective way to enhance the existing arsenal of vaccines against SARS-CoV-2 and other viruses. These adjuvants provided protective antibodies against all the tested variants of SARS-CoV-2, with only small decreases in activity against Omicron and Eta, except for the newest variants JN.1 and XBB1.16, likely due to the increased mutations in those strains. The broad, potent, and protective capacity of this adjuvant combination remains to be tested in humans; still, these results display a step forward in the implementation of adjuvants that can provide lasting and comprehensive defense to highly mutable viruses like SARS-CoV-2 and beyond.

## Electronic supplementary material

Below is the link to the electronic supplementary material.

Supplementary Material 1

## Data Availability

The data generated during the current study are available from the corresponding author on reasonable request.
