# Supplementary material for: Adjuvants to the S1-subunit of the SARS-CoV-2 spike protein vaccine improve antibody and T cell responses and surrogate neutralization in mice
Source: Sci Rep. 2024 Nov 28;14:29609. doi: 10.1038/s41598-024-80636-3 (PMC11604653; doi:10.1038/s41598-024-80636-3)
Supplement: Supplementary file 1 — Supplementary Material 1 [file 41598_2024_80636_MOESM1_ESM.docx]

**
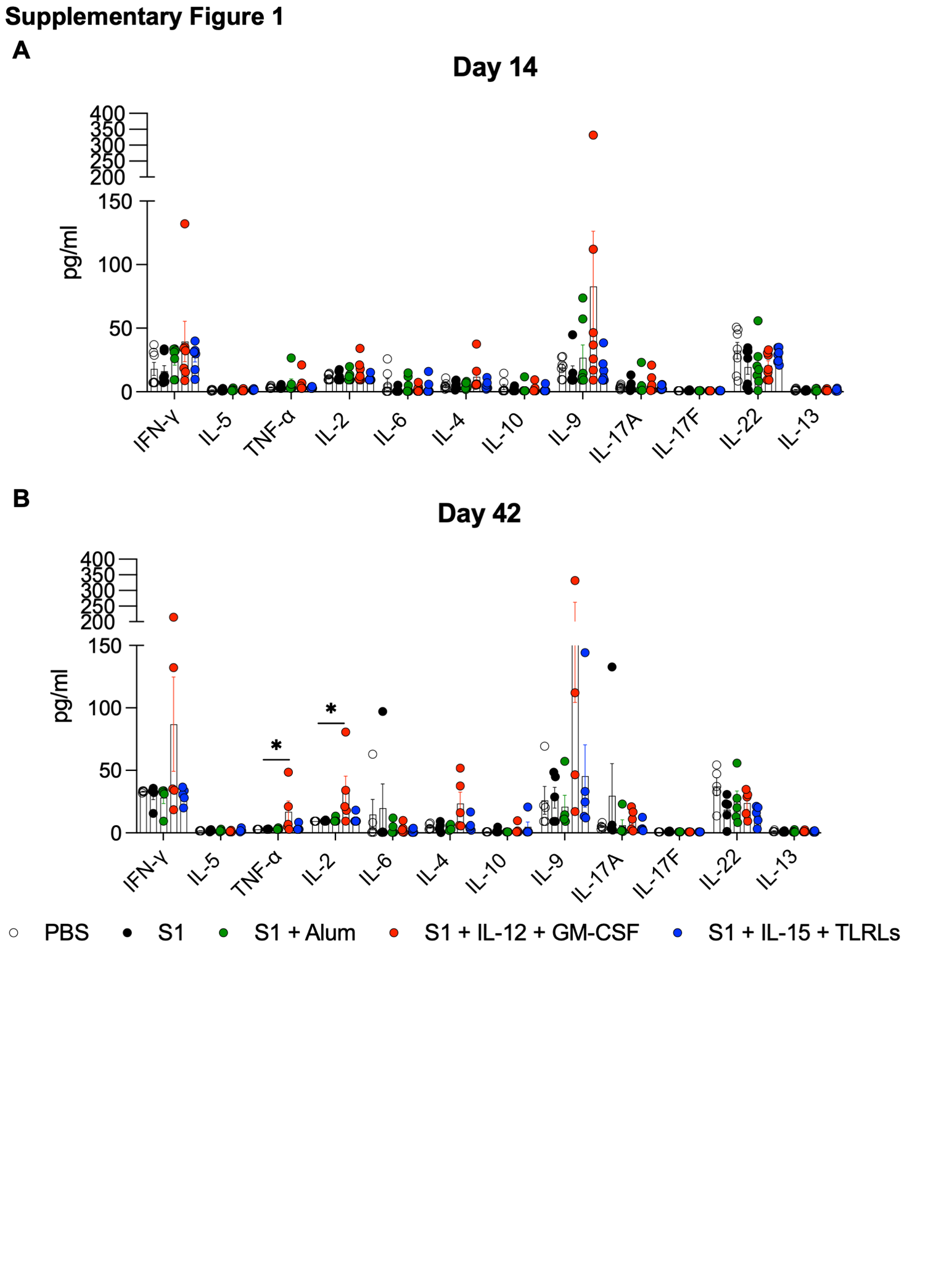
**

**Supplementary Figure 1**. B6 (WT) mice were immunized with S1 protein alone (black) or co-delivered with Alum (green), IL-12 and GM-CSF (red), or IL-15 and TLR-Ls (blue) in DOTAP. Serum samples from days 14 and 42 post-immunization were subjected to LegendPlex for detection of indicated cytokines. The data are represented as the mean ± SEM and represent one experiment. *p* values are shown using one-way ANOVA test with post hoc Tukey’s multiple comparisons; *, *p* < 0.05.

**
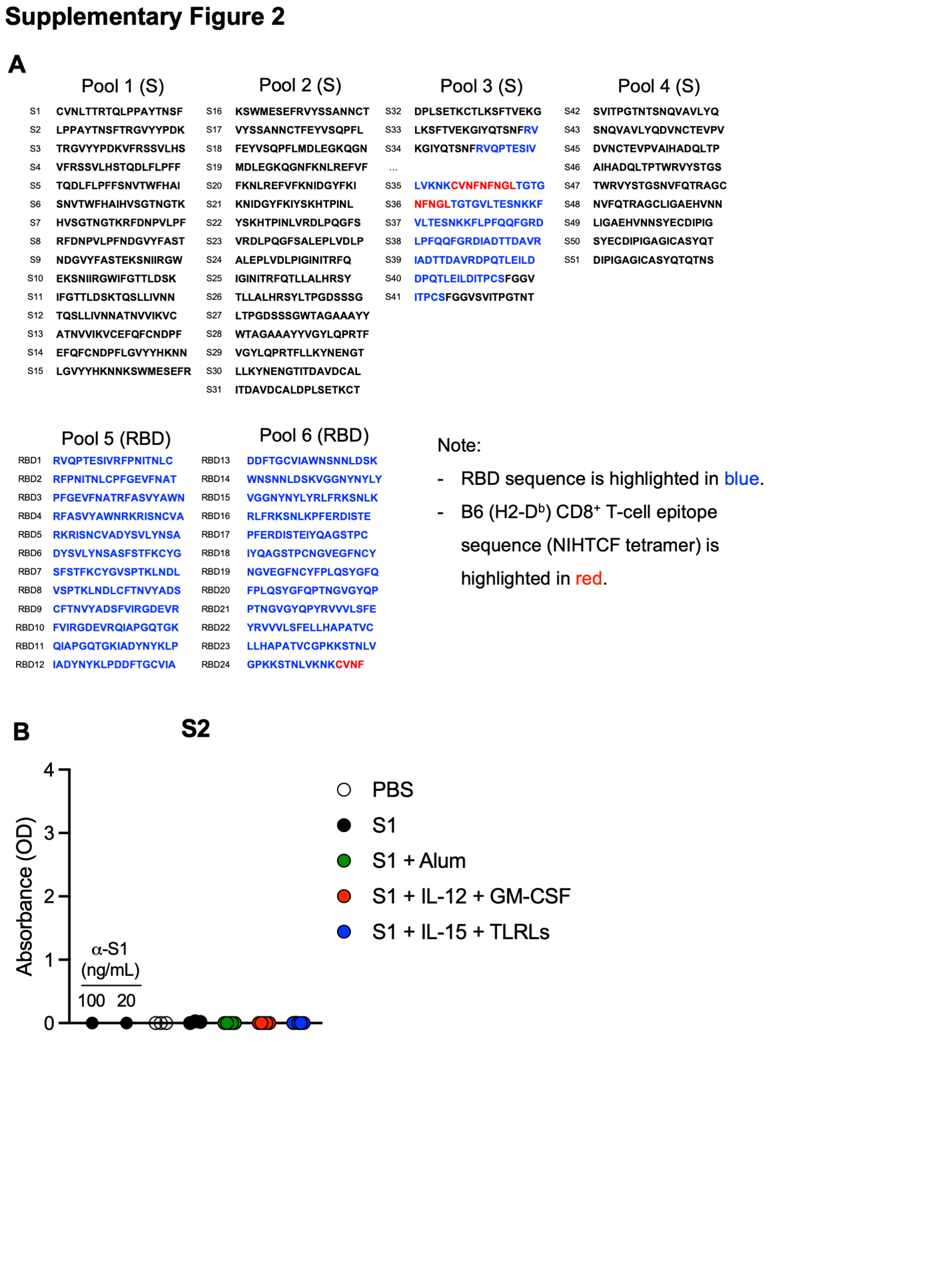
**

**Supplementary Figure 2**. Table of overlapping peptides (**A**). B6 (WT) mice were immunized with S1 protein alone (black) or co-delivered with Alum (green), IL-12 and GM-CSF (red), or IL-15 and TLR-Ls (blue) in DOTAP. Serum samples from day 60 post-immunization and 20-100 ng/ml anti-S1 antibody (open circle) or PBS as a negative control were subjected to ELISAs (read as absorbance) for cross-reactivity against S2-subunit of SARS-CoV-2 spike protein (**B**). The data are represented as individual mice. One-way ANOVA test with post hoc Tukey’s multiple comparisons was utilized; *ns*, *p* > 0.05.
